# Effectiveness of problem-based learning methodology in undergraduate medical education: a scoping review

**DOI:** 10.1186/s12909-022-03154-8

**Published:** 2022-02-17

**Authors:** Joan Carles Trullàs, Carles Blay, Elisabet Sarri, Ramon Pujol

**Affiliations:** 1 Medical Education Cathedra, School of Medicine, University of Vic-Central University of Catalonia, Vic, Barcelona, Spain; 2 Internal Medicine Service, Hospital de Olot i Comarcal de La Garrotxa, Olot, Girona, Spain; 3 The Tissue Repair and Regeneration Laboratory (TR2Lab), University of Vic-Central University of Catalonia, Vic, Barcelona, Spain; 4 Catalan Institute of Health (ICS) – Catalunya Central, Barcelona, Spain

**Keywords:** Education, Medicine, Problem-based learning, Systematic review

## Abstract

**Background:**

Problem-based learning (PBL) is a pedagogical approach that shifts the role of the teacher to the student (student-centered) and is based on self-directed learning. Although PBL has been adopted in undergraduate and postgraduate medical education, the effectiveness of the method is still under discussion. The author’s purpose was to appraise available international evidence concerning to the effectiveness and usefulness of PBL methodology in undergraduate medical teaching programs.

**Methods:**

The authors applied the Arksey and O’Malley framework to undertake a scoping review. The search was carried out in February 2021 in PubMed and Web of Science including all publications in English and Spanish with no limits on publication date, study design or country of origin.

**Results:**

The literature search identified one hundred and twenty-four publications eligible for this review. Despite the fact that this review included many studies, their design was heterogeneous and only a few provided a high scientific evidence methodology (randomized design and/or systematic reviews with meta-analysis). Furthermore, most were single-center experiences with small sample size and there were no large multi-center studies. PBL methodology obtained a high level of satisfaction, especially among students. It was more effective than other more traditional (or lecture-based methods) at improving social and communication skills, problem-solving and self-learning skills. Knowledge retention and academic performance weren’t worse (and in many studies were better) than with traditional methods. PBL was not universally widespread, probably because requires greater human resources and continuous training for its implementation.

**Conclusion:**

PBL is an effective and satisfactory methodology for medical education. It is likely that through PBL medical students will not only acquire knowledge but also other competencies that are needed in medical professionalism.

**Supplementary Information:**

The online version contains supplementary material available at 10.1186/s12909-022-03154-8.

## Background

There has always been enormous interest in identifying the best learning methods. In the mid-twentieth century, US educator Edgar Dale proposed which actions would lead to deeper learning than others and published the well-known (and at the same time controversial) “Cone of Experience or Cone of Dale”. At the apex of the cone are oral representations (verbal descriptions, written descriptions, etc.) and at the base is direct experience (based on a person carrying out the activity that they aim to learn), which represents the greatest depth of our learning. In other words, each level of the cone corresponds to various learning methods. At the base are the most effective, participative methods (what we do and what we say) and at the apex are the least effective, abstract methods (what we read and what we hear) [1]. In 1990, psychologist George Miller proposed a framework pyramid to assess clinical competence. At the lowest level of the pyramid is knowledge (knows), followed by the competence (knows how), execution (shows how) and finally the action (does) [2]. Both Miller’s pyramid and Dale’s cone propose a very efficient way of training and, at the same time, of evaluation. Miller suggested that the learning curve passes through various levels, from the acquisition of theoretical knowledge to knowing how to put this knowledge into practice and demonstrate it. Dale stated that to remember a high percentage of the acquired knowledge, a theatrical representation should be carried out or real experiences should be simulated. It is difficult to situate methodologies such as problem-based learning (PBL), case-based learning (CBL) and team-based learning (TBL) in the context of these learning frameworks.

In the last 50 years, various university education models have emerged and have attempted to reconcile teaching with learning, according to the principle that students should lead their own learning process. Perhaps one of the most successful models is PBL that came out of the English-speaking environment. There are many descriptions of PBL in the literature, but in practice there is great variability in what people understand by this methodology. The original conception of PBL as an educational strategy in medicine was initiated at McMaster University (Canada) in 1969, leaving aside the traditional methodology (which is often based on lectures) and introducing student-centered learning. The new formulation of medical education proposed by McMaster did not separate the basic sciences from the clinical sciences, and partially abandoned theoretical classes, which were taught after the presentation of the problem. In its original version, PBL is a methodology in which the starting point is a problem or a problematic situation. The situation enables students to develop a hypothesis and identify learning needs so that they can better understand the problem and meet the established learning objectives [3, 4]. PBL is taught using small groups (usually around 8–10 students) with a tutor. The aim of the group sessions is to identify a problem or scenario, define the key concepts identified, brainstorm ideas and discuss key learning objectives, research these and share this information with each other at subsequent sessions. Tutors are used to guide students, so they stay on track with the learning objectives of the task. Contemporary medical education also employs other small group learning methods including CBL and TBL. Characteristics common to the pedagogy of both CBL and TBL include the use of an authentic clinical case, active small-group learning, activation of existing knowledge and application of newly acquired knowledge. In CBL students are encouraged to engage in peer learning and apply new knowledge to these authentic clinical problems under the guidance of a facilitator. CBL encourages a structured and critical approach to clinical problem-solving, and, in contrast to PBL, is designed to allow the facilitator to correct and redirect students [5]. On the other hand, TBL offers a student-centered, instructional approach for large classes of students who are divided into small teams of typically five to seven students to solve clinically relevant problems. The overall similarities between PBL and TBL relate to the use of professionally relevant problems and small group learning, while the main difference relates to one teacher facilitating interactions between multiple self-managed teams in TBL, whereas each small group in PBL is facilitated by one teacher. Further differences are related to mandatory pre-reading assignments in TBL, testing of prior knowledge in TBL and activating prior knowledge in PBL, teacher-initiated clarifying of concepts that students struggled with in TBL versus students-generated issues that need further study in PBL, inter-team discussions in TBL and structured feedback and problems with related questions in TBL [6].

In the present study we have focused on PBL methodology, and, as attractive as the method may seem, we should consider whether it is really useful and effective as a learning method. Although PBL has been adopted in undergraduate and postgraduate medical education, the effectiveness (in terms of academic performance and/or skill improvement) of the method is still under discussion. This is due partly to the methodological difficulty in comparing PBL with traditional curricula based on lectures. To our knowledge, there is no systematic scoping review in the literature that has analyzed these aspects.

The main motivation for carrying out this research and writing this article was scientific but also professional interest. We believe that reviewing the state of the art of this methodology once it was already underway in our young Faculty of Medicine, could allow us to know if we were on the right track and if we should implement changes in the training of future doctors.

The primary goal of this study was to appraise available international evidence concerning to the effectiveness and usefulness of PBL methodology in undergraduate medical teaching programs. As the intention was to synthesize the scattered evidence available, the option was to conduct a scoping review. A scoping study tends to address broader topics where many different study designs might be applicable. Scoping studies may be particularly relevant to disciplines, such as medical education, in which the paucity of randomized controlled trials makes it difficult for researchers to undertake systematic reviews [7, 8]. Even though the scoping review methodology is not widely used in medical education, it is well established for synthesizing heterogeneous research evidence [9].

The specific aims were: 1) to determine the effectiveness of PBL in academic performance (learning and retention of knowledge) in medical education; 2) to determine the effectiveness of PBL in other skills (social and communication skills, problem solving or self-learning) in medical education; 3) to know the level of satisfaction perceived by the medical students (and/or tutors) when they are taught with the PBL methodology (or when they teach in case of tutors).

## Methods

This review was guided by Arksey and O’Malley’s methodological framework for conducting scoping reviews. The five main stages of the framework are: (1) identifying the research question; (2) ascertaining relevant studies; (3) determining study selection; (4) charting the data; and (5) collating, summarizing and reporting the results [7]. We reported our process according to the PRISMA Extension for Scoping Reviews [10].

### Stage 1: Identifying the research question

With the goals of the study established, the four members of the research team established the research questions. The primary research question was “What is the effectiveness of PBL methodology for learning in undergraduate medicine?” and the secondary question “What is the perception and satisfaction of medical students and tutors in relation to PBL methodology?”.

### Stage 2: Identifying relevant studies

After the research questions and a search strategy were defined, the searches were conducted in PubMed and Web of Science using the MeSH terms “problem-based learning” and “Medicine” (the Boolean operator “AND” was applied to the search terms). No limits were set on language, publication date, study design or country of origin. The search was carried out on 14th February 2021. Citations were uploaded to the reference manager software Mendeley Desktop (version 1.19.8) for title and abstract screening, and data characterization.

### Stage 3: Study selection

**Fig. 1:**
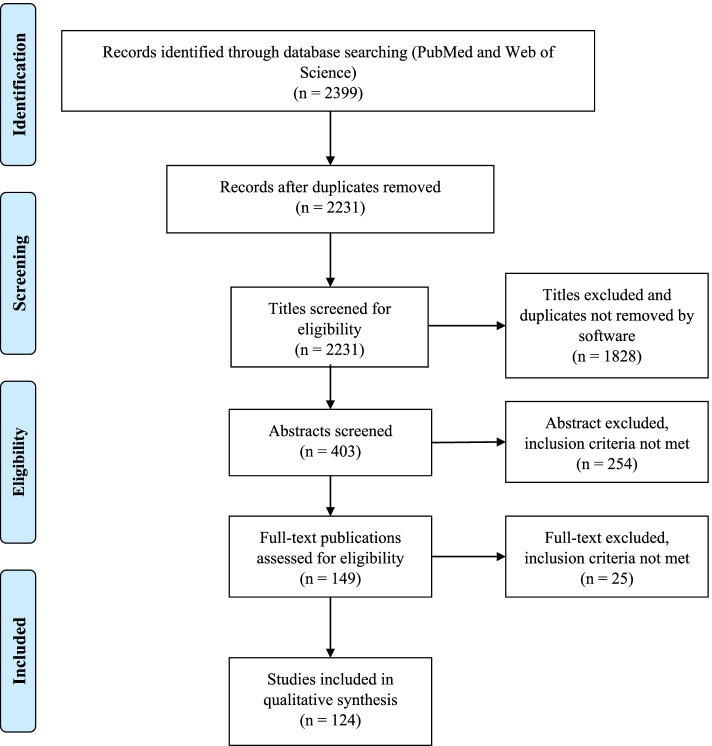
Study flow PRISMA diagram. Details the review process through the different stages of the review; includes the number of records identified, included and excluded

The searching strategy in our scoping study generated a total of 2399 references. The literature search and screening of title, abstract and full text for suitability was performed independently by one author (JCT) based on predetermined inclusion criteria. The inclusion criteria were: 1) PBL methodology was the major research topic; 2) participants were undergraduate medical students or tutors; 3) the main outcome was academic performance (learning and knowledge retention); 4) the secondary outcomes were one of the following: social and communication skills, problem solving or self-learning and/or student/tutor satisfaction; 5) all types of studies were included including descriptive papers, qualitative, quantitative and mixed studies methods, perspectives, opinion, commentary pieces and editorials. Exclusion criteria were studies including other types of participants such as postgraduate medical students, residents and other health non-medical specialties such as pharmacy, veterinary, dentistry or nursing. Studies published in languages other than Spanish and English were also excluded. Situations in which uncertainty arose, all authors (CB, ES, RP) discussed the publication together to reach a final consensus. The outcomes of the search results and screening are presented in Fig. 1. One-hundred and twenty-four articles met the inclusion criteria and were included in the final analysis.

### Stage 4: Charting the data

A data extraction table was developed by the research team. Data extracted from each of the 124 publications included general publication details (year, author, and country), sample size, study population, design/methodology, main and secondary outcomes and relevant results and/or conclusions. We compiled all data into a single spreadsheet in Microsoft Excel for coding and analysis. The characteristics and the study subject of the 124 articles included in this review are summarized in Tables 1 and 2. The detailed results of the Microsoft Excel file is also available in Additional file 1.

**Table 1:** Characteristics of the 124 publications included in the scoping review

Characteristic of the publication	Number (percentage)
**Year of publication**	
1990-1999	27 (22%)
2000-2009	46 (37%)
2010-2021	51 (41%)
**Continents and countries**^**a**^	
**Asia**	**45 (36.3%)**
China (16), Saudi Arabia (12), Egypt, India, Nepal and United Arab Emirates (2) and other^b^ nine countries (1)	
**North America**	**39 (31.5%)**
United States of America (27) and Canada (12)	
**Europe**	**22 (17.7%)**
Turkey (5), Germany and United Kingdom (4), Spain (3), France (2) and other^c^ four countries (1)	
**Africa**	**7 (5.6%)**
South Africa (4), Botswana, Ghana and Uganda (1)	
**Oceania**	**6 (4.8%)**
Australia (5) and New Zealand (1)	
**South America**	**5 (4.0%)**
México (2), Argentina, Chile and Trinidad & Tobago (1)	
**Study population**	
Students	94 (75.8%)
Students and tutors	16 (12.9%)
Tutors	6 (4.8%)
Not specified	8 (6.5%)
**Study design**	
Survey or questionnaire	45 (36.3%)
Comparative non-randomized study	32 (25.8%)
Descriptive experience	21 (16.9%)
Comparative and randomized study	16 (12.9%)
Expert opinion, editorial or comment	5 (4.0%)
Systematic review and meta-analysis	4 (3.2%)
Narrative review	1 (0.8%)
**Comparator**	
Without comparison	66 (53.2%)
With traditional or lecture-based learning	56 (45.2%)
With simulation	2 (1.6%)
**Main Outcome**^**d**^	
Performance	56 (45.2%)
Student satisfaction	36 (29.0%)
Knowledge retention	15 (12.1%)
Not specified	8 (6.5%)
Tutor satisfaction	6 (4.8%)
Other	3 (2.4%)

^a^The number of publications of each country appears in parentheses.

^b^Including: Bahrain, Iran, South Korea, Pakistan, Philippines, Singapore, Sri Lanka, Taiwan and Vietnam.

^c^Including: Belgium, Georgia, Netherlands and Sweden.

^d^Forty-eight studies included secondary outcomes: including student satisfaction (24), tutor satisfaction (9), knowledge retention (5), social and/or communication skills (5), reasoning (1) and other outcomes (4)

**Table 2:** Study design according to main and secondary outcomes and continents

	**Comparative non-randomized**	**Comparative AND randomized**	**Survey or questionnaire**	**Descriptive experience**	**Expert opinion, editorial or comment**	**Systematic review and meta-analysis**	**Narrative review**
**Number of studies**	32	16	45	21	5	4	1
**Sample size**^**a**^	162 [20–1707]	121 [31–1649]	124 [14–569]	73 [16–561]	-	1652 [1003–2061]	-
**Main outcomes**							
Performance	21	14	10	6	1	3	1
Knowledge retention	5	2	4	3	-	1	-
Student satisfaction	6	-	25	5	-	-	-
Tutor satisfaction	-	-	4	1	1	-	-
Not specified	-	-	-	5	3	-	-
Other	-	-	2	1	-	-	-
**Secondary outcomes**							
Knowledge retention	3	-	1	1	-	-	-
Student satisfaction	6	7	5	4	-	1	1
Tutor satisfaction	1	-	5	3	-	-	-
Social and/or communication skills	1	-	3	1	-	-	-
Reasoning	-	-	-	1	-	-	-
Other	2	1	-	-	-	1	-
**Continent**							
Asia	6	8	22	5	-	4	-
North America	16	2	10	9	1	-	1
Europe	4	5	7	3	3	-	-
Africa	2	-	3	2	-	-	-
Oceania	1	1	2	1	1	-	-
South America	3	-	1	1	-	-	-

^a^Sample size was available in 99 studies. Results are expressed in median and [range]

## Results

### Stage 5: Collating, summarizing and reporting the results

As indicated in the search strategy (Fig. 1) this review resulted in the inclusion of 124 publications. Publication years of the final sample ranged from 1990 to 2020, the majority of the publications (51, 41%) were identified for the years 2010–2020 and the years in which there were more publications were 2001, 2009 and 2015. Countries from the six continents were represented in this review. Most of the publications were from Asia (especially China and Saudi Arabia) and North America followed by Europe, and few studies were from Africa, Oceania and South America. The country with more publications was the United States of America (*n* = 27). The most frequent designs of the selected studies were surveys or questionnaires (*n* = 45) and comparative studies (*n* = 48, only 16 were randomized) with traditional or lecture-based learning methodologies (in two studies the comparison was with simulation) and the most frequently measured outcomes were academic performance followed by student satisfaction (48 studies measured more than one outcome). The few studies with the highest level of scientific evidence (systematic review and meta-analysis and randomized studies) were conducted mostly in Asian countries (Tables 1 and 2). The study subject was specified in 81 publications finding a high variability but at the same time great representability of almost all disciplines of the medical studies.

The sample size was available in 99 publications and the median [range] of the participants was 132 [14–2061]. According to study population, there were more participants in the students’ focused studies (median 134 and range 16–2061) in comparison with the tutors’ studies (median 53 and range 14–494).

Finally, after reviewing in detail the measured outcomes (main and secondary) according to the study design (Table 2 and Additional file 1) we present a narrative overview and a synthesis of the main findings.

### Main outcome: academic performance (learning and knowledge retention)

Seventy-one of the 124 publications had learning and/or knowledge retention as a measured outcome, most of them (*n* = 45) were comparative studies with traditional or lecture-based learning and 16 were randomized. These studies were varied in their methodology, were performed in different geographic zones, and normally analyzed the experience of just one education center. Most studies (*n* = 49) reported superiority of PBL in learning and knowledge acquisition [11–59] but there was no difference between traditional and PBL curriculums in another 19 studies [60–78]. Only three studies reported that PBL was less effective [79–81], two of them were randomized (in one case favoring simulation-based learning [80] and another favoring lectures [81]) and the remaining study was based on tutors’ opinion rather than real academic performance [79]. It is noteworthy that the four systematic reviews and meta-analysis included in this scoping review, all carried out in China, found that PBL was more effective than lecture-based learning in improving knowledge and other skills (clinical, problem-solving, self-learning and collaborative) [40, 51, 53, 58]. Another relevant example of the superiority of the PBL method over the traditional method is the experience reported by Hoffman et al. from the University of Missouri-Columbia. The authors analyzed the impact of implementing the PBL methodology in its Faculty of Medicine and revealed an improvement in the academic results that lasted for over a decade [31].

### Secondary outcomes

#### Social and communication skills

We found five studies in this scoping review that focused on these outcomes and all of them described that a curriculum centered on PBL seems to instill more confidence in social and communication skills among students. Students perceived PBL positively for teamwork, communication skills and interpersonal relations [44, 45, 67, 75, 82].

#### Student satisfaction

Sixty publications analyzed student satisfaction with PBL methodology. The most frequent methodology were surveys or questionnaires (30 studies) followed by comparative studies with traditional or lecture-based methodology (19 studies, 7 of them were randomized). Almost all the studies (51) have shown that PBL is generally well-received [11, 13, 18–22, 26, 29, 34, 37, 39, 41, 42, 46, 50, 56, 58, 63, 64, 66, 78, 82–110] but in 9 studies the overall satisfaction scores for the PBL program were neutral [76, 111–116] or negative [117, 118]. Some factors that have been identified as key components for PBL to be successful include: a small group size, the use of scenarios of realistic cases and good management of group dynamics. Despite a mostly positive assessment of the PBL methodology by the students, there were some negative aspects that could be criticized or improved. These include unclear communication of the learning methodology, objectives and assessment method; bad management and organization of the sessions; tutors having little experience of the method; and a lack of standardization in the implementation of the method by the tutors.

#### Tutor satisfaction

There are only 15 publications that analyze the satisfaction of tutors, most of them surveys or questionnaires [85, 88, 92, 98, 108, 110, 119]. In comparison with the satisfaction of the students, here the results are more neutral [112, 113, 115, 120, 121] and even unfavorable to the PBL methodology in two publications [117, 122]. PBL teaching was favored by tutors when the institutions train them in the subject, when there was administrative support and adequate infrastructure and coordination [123]. In some experiences, the PBL modules created an unacceptable toll of anxiety, unhappiness and strained relations.

#### Other skills (problem solving and self-learning)

The effectiveness of the PBL methodology has also been explored in other outcomes such as the ability to solve problems and to self-directed learning. All studies have shown that PBL is more effective than lecture-based learning in problem-solving and self-learning skills [18, 24, 40, 48, 67, 75, 93, 104, 124]. One single study found a poor accuracy of the students’ self-assessment when compared to their own performance [125]. In addition, there are studies that support PBL methodology for integration between basic and clinical sciences [126].

Finally, other publications have reported the experience of some faculties in the implementation of the PBL methodology. Different experiences have demonstrated that it is both possible and feasible to shift from a traditional curriculum to a PBL program, recognizing that PBL methodology is complex to plan and structure, needs a large number of human and material resources, requiring an immense teacher effort [28, 31, 94, 127–133]. In addition, and despite its cost implication, a PBL curriculum can be successfully implemented in resource-constrained settings [134, 135].

## Discussion

We conducted this scoping review to explore the effectiveness and satisfaction of PBL methodology for teaching in undergraduate medicine and, to our knowledge, it is the only study of its kind (systematic scoping review) that has been carried out in the last years. Similarly, Vernon et al. conducted a meta-analysis of articles published between 1970 and 1992 and their results generally supported the superiority of the PBL approach over more traditional methods of medical education [136]. PBL methodology is implemented in medical studies on the six continents but there is more experience (or at least more publications) from Asian countries and North America. Despite its apparent difficulties on implementation, a PBL curriculum can be successfully implemented in resource-constrained settings [134, 135]. Although it is true that the few studies with the highest level of scientific evidence (randomized studies and meta-analysis) were carried out mainly in Asian countries (and some in North America and Europe), there were no significant differences in the main results according to geographical origin.

In this scoping review we have included a large number of publications that, despite their heterogeneity, tend to show favorable results for the usefulness of the PBL methodology in teaching and learning medicine. The results tend to be especially favorable to PBL methodology when it is compared with traditional or lecture-based teaching methods, but when compared with simulation it is not so clear. There are two studies that show neutral [71] or superior [80] results to simulation for the acquisition of specific clinical skills. It seems important to highlight that the four meta-analysis included in this review, which included a high number of participants, show results that are clearly favorable to the PBL methodology in terms of knowledge, clinical skills, problem-solving, self-learning and satisfaction [40, 51, 53, 58].

Regarding the level of satisfaction described in the surveys or questionnaires, the overall satisfaction rate was higher in the PBL students when compared with traditional learning students. Students work in small groups, allowing and promoting teamwork and facilitating social and communication skills. As sessions are more attractive and dynamic than traditional classes, this could lead to a greater degree of motivation for learning.

These satisfaction results are not so favorable when tutors are asked and this may be due to different reasons; first, some studies are from the 90s, when the methodology was not yet fully implemented; second, the number of tutors included in these studies is low; and third, and perhaps most importantly, the complaints are not usually due to the methodology itself, but rather due to lack of administrative support, and/or work overload. PBL methodology implies more human and material resources. The lack of experience in guided self-learning by lecturers requires more training. Some teachers may not feel comfortable with the method and therefore do not apply it correctly.

Despite how effective and/or attractive the PBL methodology may seem, some (not many) authors are clearly detractors and have published opinion articles with fierce criticism to this methodology. Some of the arguments against are as follows: clinical problem solving is the wrong task for preclinical medical students, self-directed learning interpreted as self-teaching is not appropriate in undergraduate medical education, relegation to the role of facilitators is a misuse of the faculty, small-group experience is inherently variable and sometimes dysfunctional, etc. [137].

In light of the results found in our study, we believe that PBL is an adequate methodology for the training of future doctors and reinforces the idea that the PBL should have an important weight in the curriculum of our medical school. It is likely that training through PBL, the doctors of the future will not only have great knowledge but may also acquire greater capacity for communication, problem solving and self-learning, all of which are characteristics that are required in medical professionalism. For this purpose, Koh et al. analyzed the effect that PBL during medical school had on physician competencies after graduation, finding a positive effect mainly in social and cognitive dimensions [138].

Despite its defects and limitations, we must not abandon this methodology and, in any case, perhaps PBL should evolve, adapt, and improve to enhance its strengths and improve its weaknesses. It is likely that the new generations, trained in schools using new technologies and methodologies far from lectures, will feel more comfortable (either as students or as tutors) with methodologies more like PBL (small groups and work focused on problems or projects). It would be interesting to examine the implementation of technologies and even social media into PBL sessions, an issue that has been poorly explorer [139].

### Limitations

Scoping reviews are not without limitations. Our review includes 124 articles from the 2399 initially identified and despite our efforts to be as comprehensive as possible, we may have missed some (probably few) articles. Even though this review includes many studies, their design is very heterogeneous, only a few include a large sample size and high scientific evidence methodology. Furthermore, most are single-center experiences and there are no large multi-center studies. Finally, the frequency of the PBL sessions (from once or twice a year to the whole curriculum) was not considered, in part, because most of the revised studies did not specify this information. This factor could affect the efficiency of PBL and the perceptions of students and tutors about PBL. However, the adoption of a scoping review methodology was effective in terms of summarizing the research findings, identifying limitations in studies’ methodologies and findings and provided a more rigorous vision of the international state of the art.

## Conclusions

This systematic scoping review provides a broad overview of the efficacy of PBL methodology in undergraduate medicine teaching from different countries and institutions. PBL is not a new teaching method given that it has already been 50 years since it was implemented in medicine courses. It is a method that shifts the leading role from teachers to students and is based on guided self-learning. If it is applied properly, the degree of satisfaction is high, especially for students. PBL is more effective than traditional methods (based mainly on lectures) at improving social and communication skills, problem-solving and self-learning skills, and has no worse results (and in many studies better results) in relation to academic performance. Despite that, its use is not universally widespread, probably because it requires greater human resources and continuous training for its implementation. In any case, more comparative and randomized studies and/or other systematic reviews and meta-analysis are required to determine which educational strategies could be most suitable for the training of future doctors.

## Supplementary Information

**Additional file 1.** Characteristics ofthe 124 included studies.

## Abbreviations

PBL: Problem-based learning
CBL: Case-based learning
TBL: Team-based learning

## Declarations

### Availability of data and materials

The datasets used and/or analyzed during the current study are available from the corresponding author on reasonable request.
