# Supplementary material for: Effectiveness of problem-based learning methodology in undergraduate medical education: a scoping review
Source: BMC Med Educ. 2022 Feb 17;22:104. doi: 10.1186/s12909-022-03154-8 (PMC8851721; doi:10.1186/s12909-022-03154-8)
Supplement: Supplementary file 1 — Additional file 1. Characteristics ofthe 124 included studies. [file 12909_2022_3154_MOESM1_ESM.docx]

**Additional file 1. Characteristics of the 124 included studies**

| **Year** | **Author** | **Country** | **Continent** | **Sample size** | **Study population** | **Design /Methodology** | **Comparator** | **Study subject** | **Outcome** | **Outcome 2** | Main results |
| --- | --- | --- | --- | --- | --- | --- | --- | --- | --- | --- | --- |
| 1990 | Sokas | United States of America | America (north) | 69 | Students | Comparative non-randomized study | Traditional or lecture | Occupational health | Performance | Student satisfaction | PBL with specific occupational content is well accepted by students and modestly improves their occupational history taking |
| 1991 | Blosser | United States of America | America (north) | - | Students | Descriptive experience | None | Surgery | Student satisfaction | - | Students found the PBL component of the clerkship highly motivating, intellectually stimulating, and experientially satisfying |
| 1991 | Usherwood | United Kingdom | Europe | 53 | Students | Survey or questionnaire | None | General practice and public health medicine | Student satisfaction | - | The objectives of the program were achieved, students were provided with a flexible environment within which to learn about general practice and public health and acquiring skills of self-assessment and self-directed learning |
| 1992 | Schwartz | United States of America | America (north) | 57 | Students | Comparative non-randomized study | Traditional or lecture | Surgery | Knowledge retention | Other | PBL is similar to a traditional curricular format in improving students’ knowledge and superior in improving clinical problem solving skills |
| 1993 | Mennin | United States of America | America (north) | 1649 | Students | Comparative and randomized study | Traditional or lecture | The global curriculum | Performance | - | In the short run the conventional curriculum better prepared the students. In the long run the PBL curriculum better prepared the students |
| 1993 | Des Marchais | Canada | America (north) | - | Not specified | Descriptive experience | None | The global curriculum | Not specified | - | The experience demonstrated that it is both possible and feasible to shift from a traditional curriculum to a PBL and community-oriented program |
| 1995 | Chang | Canada | America (north) | - | Students | Narrative review | Traditional or lecture | Surgery | Not specified | - | Comparable examination results between PBL and traditional. More satisfied with PBL |
| 1995 | Berntein | Canada | America (north) | 265 | Students and tutors | Comparative non-randomized study | Traditional or lecture | Not specified | Student satisfaction | Tutor satisfaction | The experience with PBL led to more favorable attitudes among the students and faculty |
| 1996 | Kaufman | Canada | America (north) | 168 | Students | Comparative non-randomized study | Traditional or lecture | The global curriculum | Student satisfaction | - | Superiority of the PBL curriculum regarding the students attitudes toward their medical education |
| 1996 | Richards | United States of America | America (north) | 452 | Students | Comparative non-randomized study | Traditional or lecture | Internal Medicine | Performance | - | A PBL curricula may enhance students clinical performance |
| 1996 | Kalaian | United States of America | America (north) | 172 | Students | Survey or questionnaire | None | Infectious Diseases and Cardiovascular | Student satisfaction | - | In careful constructed PBL curriculum students may be able to develop and sustain skills |
| 1996 | Gresham | United States of America | America (north) | 64 | Students | Descriptive experience | None | Not specified | Performance | Student satisfaction | PBL strategies are well suited to clinical education provided that the faculty are committed to this methodology. It appears to foster the development of learning skills |
| 1996 | Griffith | United States of America | America (north) | 53 | Tutors | Survey or questionnaire | None | Internal Medicine | Tutor satisfaction | - | Tutors believed that the new curriculum interfered with student participation on the ward team and their ability to teach students. Students learn clinical medicine best on ward teams seeing patients rather than through PBL |
| 1996 | De Lowerntal | South Africa | Africa | - | Students and tutors | Descriptive experience | None | Not specified | Tutor satisfaction | Student satisfaction | The module created an unacceptable toll of anxiety, unhappiness and strained relations. The latter may have contributed to the subsequent rejection of a proposal for a faculty-wide PBL curriculum by members of the Faculty |
| 1996 | Vernon | United States of America | America (north) | 494 | Tutors | Survey or questionnaire | None | Not specified | Performance | Knowledge retention | The value of PBL was judged to be high with respect to student interest, reasoning and clinical preparation and low with respect to learning efficiency and basic science knowledge |
| 1997 | Vincelette | Canada | America (north) | - | Students and tutors | Descriptive experience | None | The global curriculum | Student satisfaction | Tutor satisfaction | A pilot PBL program was well received by both the faculty and the students |
| 1998 | Kaufman | Canada | America (north) | 243 | Students | Comparative non-randomized study | Traditional or lecture | The global curriculum | Performance | - | PBL and conventional graduates performed similarly except in psychiatry, preventive medicine and public health were PBL graduates scored higher |
| 1998 | Hill | Australia | Oceania | 139 | Students | Comparative non-randomized study | Traditional or lecture | Not specified | Performance | - | PBL medical school graduates rated highly than traditional graduates in interpersonal skills, confidence, collaboration, holistic care and self-directed learning |
| 1998 | Blake | United States of America | America (north) | 41 | Tutors | Survey or questionnaire | None | Not specified | Performance | - | Evaluators had a favorable perception of the clinical performance of the first cohort of students to complete a new preclinical curriculum that had PBL as a central component |
| 1998 | Hmelo | United States of America | America (north) | 76 | Students | Comparative non-randomized study | Traditional or lecture | Not specified | Knowledge retention | | There are important cognitive benefits of the PBL approach |
| 1999 | Kaufman | Canada | America (north) | 243 | Students | Comparative non-randomized study | Traditional or lecture | The global curriculum | Performance | Student satisfaction | Performance of PBL and conventional classes is equivalent after medical school, and during postgraduate education. PBL students report greater satisfaction |
| 1999 | Antepohl | Germany | Europe | 123 | Students | Comparative and randomized study | Traditional or lecture | Pharmacology | Performance | Student satisfaction | Similar scores with a tendency favoring PBL students. Students considered PBL to be a more effective and satisfactory method |
| 1999 | Finch | Canada | America (north) | 47 | Students | Comparative non-randomized study | Traditional or lecture | Pediatric | Performance | Knowledge retention | PBL students had more knowledge and higher cognitive-related patients management skills |
| 1999 | McGrew | United States of America | America (north) | - | Students and tutors | Survey or questionnaire | None | Family Medicine | Not specified | - | Students and faculty ranked PBL sessions higher than any other nonclinical component of the clerkship |
| 1999 | Casassus | France | Europe | 68 | Students | Comparative non-randomized study | Traditional or lecture | Hematology | Performance | Student satisfaction | PBL students performed better in their clinical problem-solving examinations than did students from traditional methods. They were also more satisfied |
| 1999 | Purdy | Canada | America (north) | - | Students | Descriptive experience | None | Neurosciences | Performance | Student satisfaction | Students' performance has been excellent and they have viewed the unit as stimulating and enjoyable. Faculty involved as tutors also have been delighted with their experience |
| 1999 | Farrell | United States of America | America (north) | 75 | Students | Survey or questionnaire | None | Ophthalmology | Performance | Student satisfaction | The PBL program showed significant knowledge gains and high student satisfaction |
| 2000 | Kelly | Australia | Oceania | - | Students | Descriptive experience | None | Emergency Medicine | Not specified | - | Feedback from students was very positive about format, content and usefulness in learning |
| 2000 | Doig | United States of America | America (north) | - | Not specified | Descriptive experience | None | The global curriculum | Not specified | - | The curricular structure was successful in improving students’ perceptions of and performance in basic sciences and promotes solid knowledge construction |
| 2000 | Ghosh | Nepal | Asia | 100 | Students | Survey or questionnaire | None | Physiology | Student satisfaction | - | The majority of the students opined that the combination of didactic lectures and PBL sessions was definitely beneficial regarding all the aspects of learning |
| 2001 | Bui-Mansfield | United States of America | America (north) | 76 | Students and tutors | Descriptive experience | None | Radiology | Not specified | - | Both, students and tutors were satisfied with the PBL experience |
| 2001 | Dyke | Australia | Oceania | 136 | Students | Comparative and randomized study | Traditional or lecture | Epidemiology | Performance | Student satisfaction | PBL provides an academically equivalent but personally far richer learning experience |
| 2001 | Brewer | United States of America | America (north) | - | Students | Descriptive experience | Traditional or lecture | Endocrinology | Performance | - | Differences in students’ performance were small |
| 2001 | Walters | United States of America | America (north) | - | Students | Survey or questionnaire | None | Endocrinology | Student satisfaction | - | Student ratings were highly successful |
| 2001 | Leung | China | Asia | 320 | Students | Survey or questionnaire | None | Public Health | Student satisfaction | - | Eighty-eight percent of students found the PBL module useful or very useful |
| 2001 | Curtis | United States of America | America (north) | 639 | Students | Comparative non-randomized study | Traditional or lecture | Pediatric | Knowledge retention | Student satisfaction | PBL was associated with higher scores on the examinations and increased student satisfaction |
| 2001 | Seneviratne | Sri Lanka | Asia | 188 | Students | Survey or questionnaire | None | Not specified | Performance | Communication skills | Educational advantage of PBL were high in communication and problem-solving skills. The main disadvantage was that it was time-consuming |
| 2001 | Khoo | Singapore | Asia | - | Students and tutors | Survey or questionnaire | None | Not specified | Student satisfaction | Tutor satisfaction | Overall, the experiences were positive and both groups are willing to "struggle" with this new way of learning |
| 2001 | Villamor | Philippines | Asia | 68 | Students | Survey or questionnaire | None | Biochemistry of the endocrine system | Student satisfaction | - | PBL approach motivated students to acquire self-learning skills. Students found that PBL approach inspired them to take charge of their own learning |
| 2002 | Trevena | Australia | Oceania | 130 | Students | Survey or questionnaire | None | Public Health | Performance | Student satisfaction | High level of satisfaction and competence |
| 2002 | Alleyne | Trinidad and Tobago | America (south) | 129 | Students | Comparative non-randomized study | Traditional or lecture | Medicine, Surgery and Obstetrics and Gynecology | Performance | - | There were no significant differences in the performance of the two groups of students |
| 2002 | Brynhildsen | Sweden | Europe | 208 | Students and tutors | Survey or questionnaire | None | Not specified | Other | - | Both students and teachers support PBL for integration between basic and clinical sciences |
| 2002 | Astin | United Kingdom | Europe | - | Students | Opinion, editorial, comment | None | Medical Statistics | Performance | - | Data interpretation and objective structured clinical examinations are more productive with PBL than traditional teaching |
| 2002 | Whitfield | United States of America | America (north) | 617 | Students | Comparative non-randomized study | Traditional or lecture | Not specified | Performance | Knowledge retention | PBL effect size on students’ scores for fund of knowledge and clinical problem-solving skills was small to moderate |
| 2002 | Tousignant | Canada | America (north) | 70 | Students | Survey or questionnaire | None | Not specified | Other | - | The students in the third year of a self-directed PBL medical four year program demonstrated poor accuracy of the self-assessment when compared to their own performance |
| 2003 | Carrera | Argentina | America (south) | - | Not specified | Descriptive experience | None | Not specified | Not specified | - | Schools in developing countries should consider whether a PBL curriculum is appropriate and should realize the difficulties they will have to overcome |
| 2004 | Chang | China | Asia | 137 | Students | Comparative non-randomized study | Traditional or lecture | Anesthesia | Student satisfaction | - | Implementation of PBL showed satisfactory results. Students preferred PBL over lecture-based traditional teaching in 6 (out of 8) items |
| 2004 | McParland | United Kingdom | Europe | 379 | Students | Comparative non-randomized study | Traditional or lecture | Psychiatry | Performance | - | The PBL curriculum resulted in significantly better examination performance than did the traditional teaching curriculum |
| 2004 | Subramaniam | New Zealand | Oceania | - | Tutors | Survey or questionnaire | None | Radiology | Tutor satisfaction | - | 50% of the radiologists who teach using PBL methods expressed that PBL is a better method for radiology teaching than traditional methods |
| 2004 | McLean | South Africa | Africa | 20 | Students | Comparative non-randomized study | Traditional or lecture | Not specified | Student satisfaction | - | PBL students are generally more content with their studies than their conventional curriculum counterparts |
| 2005 | Casey | United States of America | America (north) | 162 | Students | Comparative non-randomized study | Traditional or lecture | Obstetrics and gynecology | Performance | Student satisfaction | Students’ satisfaction and performance improved significantly when PBL methods were introduced |
| 2005 | Gurpinar | Turkey | Europe | 134 | Students | Comparative non-randomized study | Traditional or lecture | Public Health | Knowledge retention | | There was a statistically significant difference between knowledge scores of PBL and Traditional education groups in favor of the PBL group |
| 2005 | Tamblyn | Canada | America (north) | 751 | Students | Comparative non-randomized study | Traditional or lecture | Preventive Care | Performance | - | Transition to a PBL curriculum was associated with significant improvements in preventive care, continuity of care and indicators of diagnostic performance |
| 2005 | Abu-Hijleh | Bahrain | Asia | 131 | Students | Survey or questionnaire | None | Surgery | Performance | Student satisfaction | Students perceived the course positively and had a positive impact on their performance in the final qualifying examination |
| 2005 | Kemahli | Turkey | Europe | - | Not specified | Opinion, editorial, comment | None | Hematology | Not specified | - | PBL is a powerful learning tool and can be used as the major method in preclinical medical education and no hematology topic remains omitted |
| 2005 | Salinas Sánchez | Spain | Europe | - | Not specified | Opinion, editorial, comment | None | Urology | Not specified | - | PBL methodology is complex to plan and structure, needs a large number of human and material resources, requiring an immense teacher effort |
| 2005 | Distlehorst | United States of America | America (north) | 648 | Students | Comparative non-randomized study | Traditional or lecture | The global curriculum | Performance | Knowledge retention | In several performance measures the PBL students performed significantly better, and in no circumstance performed worse than the traditional students |
| 2005 | Grkoviæ | Australia | Oceania | - | Not specified | Opinion, editorial, comment | None | Not specified | Not specified | - | There are multiple benefits of curriculum reform leading towards the PBL approach. The authors experience was very positive |
| 2006 | Lucas | Spain | Europe | - | Students | Survey or questionnaire | None | Anesthesia | Student satisfaction | - | The students' level of satisfaction with and acceptance of PBL were high |
| 2006 | Burgun | France | Europe | 177 | Students | Survey or questionnaire | None | Medical informatics | Student satisfaction | - | PBL to teach information and communication technology is feasible. The overall opinion of the students was good responding positively to the program |
| 2006 | Steadman | United States of America | America (north) | 31 | Students | Comparative and randomized study | Simulation | Critical care skills | Performance | - | Simulation-based learning was superior to PBL for the acquisition of critical assessment and management skills |
| 2006 | Hoffman | United States of America | America (north) | - | Students | Comparative non-randomized study | Traditional or lecture | The global curriculum | Performance | - | Changing from a traditional to a PBL curriculum improved the graduates in knowledge and skills needed to clinical practice. PBL was an effective learning strategy in the medical school curriculum |
| 2008 | Norman | Canada | America (north) | 1166 | Students | Comparative non-randomized study | Traditional or lecture | Not specified | Performance | - | There is no evidence that PBL graduates are better to maintain competence than graduates of conventional schools |
| 2008 | Cohen-Schotanus | Netherlands | Europe | 344 | Students | Comparative and randomized study | Traditional or lecture | Not specified | Performance | Other | The results add further validity with respect to self-rated competencies in favor of PBL. No differences were found on clinical competence and career development |
| 2009 | Wenk | Germany | Europe | 33 | Students | Comparative and randomized study | Simulation | Anesthesia | Performance | - | Both PBL and simulation lead to comparable short-term outcomes in theoretical knowledge and clinical skills |
| 2009 | Kong | China | Asia | 90 | Students | Comparative and randomized study | Traditional or lecture | Ophthalmology | Performance | - | The PBL groups had significantly higher mean results of theoretical and case analysis examinations |
| 2009 | Collard | Belgium | Europe | 104 | Students | Descriptive experience | None | Endocrinology | Knowledge retention | Reasoning | Reasoning skills are evidenced early in a curriculum involving PBL and increase during training. This is accompanied by a decrease in knowledge retention |
| 2009 | Johnston | China | Asia | 129 | Students | Comparative and randomized study | Traditional or lecture | Evidence-based medicine | Performance | - | PBL was less effective at imparting knowledge than the usual teaching program of a lecture followed by a group tutorial |
| 2009 | Macallan | United Kingdom | Europe | - | Students and tutors | Descriptive experience | None | Not specified | Student satisfaction | Tutor satisfaction | Students found PBL a positive learning experience. PBL is a parallel teaching approach that helps structure the teaching week, but does not replace traditional bedside teaching |
| 2009 | Gurpinar | Turkey | Europe | 323 | Students and tutors | Survey or questionnaire | None | Not specified | Student satisfaction | Tutor satisfaction | PBL is well received by tutors and students. PBL offers significant contribution to the students in areas that are considered to be superior aspects of PBL when compared to conventional education |
| 2009 | Tsou | China | Asia | 71 | Students | Descriptive experience | None | Not specified | Performance | Knowledge retention | A PBL curriculum is feasible and encourage students to improve self-directed learning, learn adequate knowledge in basic sciences, and experience positive effects on learning clinical medicine |
| 2009 | Lin | China | Asia | - | Students and tutors | Descriptive experience | None | Not specified | Not specified | - | The medical curriculum has undergone a substantial change to incorporate PBL into the curriculum. Despite ongoing improvements, there are still many problems |
| 2009 | Tufts | South Africa | Africa | 569 | Students | Survey or questionnaire | None | Physiology | Student satisfaction | - | Greater interaction of students with experts is needed. In particular, students felt that they lacked the basic conceptual foundations essential for the learning and understanding of physiology |
| 2010 | Suleman | Saudi Arabia | Asia | 54 | Students | Comparative non-randomized study | Traditional or lecture | Not specified | Student satisfaction | Communication skills | PBL was perceived as better learning method in 13 aspects, especially in enhancing team work and communication skills |
| 2010 | Wang | China | Asia | 173 | Students | Comparative non-randomized study | Traditional or lecture | Anatomy | Performance | Student satisfaction | The study evidences that the PBL approach stimulates students’ interest in learning and enhances anatomy education |
| 2011 | Abou-Elhamd | Egypt | Asia | - | Not specified | Descriptive experience | None | Otolaryngology | Knowledge retention | | The application of PBL to ENT teaching has resulted in a substantial increase in students’ knowledge |
| 2011 | Urrutia | Mexico | America (south) | 340 | Students | Comparative non-randomized study | Traditional or lecture | Not specified | Knowledge retention | Other | PBL influenced in a positive and significant way the students’ perception to solve problems and their motivation to achieve a good academic performance |
| 2012 | Nouns | Germany | Europe | 240 | Students | Comparative and randomized study | Traditional or lecture | Basic medical sciences | Knowledge retention | | There is no difference between a traditional and a PBL curriculum in terms of the assimilation of overall medical knowledge |
| 2012 | Tian | China | Asia | 107 | Students | Comparative and randomized study | Traditional or lecture | Evidence-based medicine | Performance | Student satisfaction | The PBL program was more effective (in both performance and student satisfaction) than the lecture-based program |
| 2012 | Elzubeir | Saudi Arabia | Asia | 20 | Students and tutors | Survey or questionnaire | None | Renal system | Student satisfaction | - | Students’ and tutors had favorable perceptions of amount learned and stimulation |
| 2012 | Saloojee | South Africa | Africa | 1707 | Students | Comparative non-randomized study | Traditional or lecture | Psychiatry | Performance | - | PBL had a positive effect on students’ ability to solve problems based on written case scenarios, but no striking difference in their knowledge or clinical skills |
| 2012 | Hoover | United States of America | America (north) | 16 | Students | Descriptive experience | None | Public Health | Performance | - | PBL is a promising tool to enhance medical students’ engagement with public health |
| 2013 | Li | China | Asia | 120 | Students | Comparative and randomized study | Traditional or lecture | Dermatology | Performance | Student satisfaction | All PBL participants had better results for written examination, clinical examination and overall performance and also greater satisfaction |
| 2013 | Sulaiman | United Arab Emirates | Asia | 217 | Students and tutors | Survey or questionnaire | None | Family Medicine | Student satisfaction | - | Students and tutors highly rated the PBL program as a valuable learning methodology that enhanced knowledge and understanding patient problems |
| 2013 | Albarrak | Saudi Arabia | Asia | 200 | Students | Survey or questionnaire | Traditional or lecture | Not specified | Student satisfaction | - | The overall satisfaction rate was higher in the PBL students when compared with traditional learning students |
| 2014 | Ding | China | Asia | 2061 | Students | Systematic review and meta-analysis | Traditional or lecture | Preventive Medicine | Performance | Other | PBL was more effective than lecture-based learning in improving knowledge, attitude and other skills (problem-solving, self-learning and collaborative). |
| 2014 | Navarro | Chile | America (south) | 14 | Tutors | Survey or questionnaire | None | Not specified | Tutor satisfaction | - | PBL teaching is favored by tutors when the institutions train them in the subject, when there is administrative support and adequate infrastructure and coordination |
| 2014 | Meo | Saudi Arabia | Asia | 60 | Students | Survey or questionnaire | Traditional or lecture | Respiratory | Knowledge retention | Student satisfaction | Students with PBL curriculum had more positive perceptions on learning, knowledge and skills and satisfaction compared to the students belonging to the traditional style |
| 2014 | Khoshnevisasl | Iran | Asia | 40 | Students | Comparative and randomized study | Traditional or lecture | Pediatric | Performance | Student satisfaction | Scores were higher in PBL compared to lecture group. Students preferred PBL because of motivation, higher quality of education, knowledge retention, class attractiveness, and practical use |
| 2015 | Grisham | Vietnam | Asia | 61 | Students and tutors | Survey or questionnaire | None | Public Health | Student satisfaction | - | Students and tutors regarded PBL positively. However, there was consensus that hybrid models are probably the most beneficial for public health education |
| 2015 | Aboonq | Saudi Arabia | Asia | 110 | Tutors | Survey or questionnaire | None | Not specified | Other | - | Considerably high proportion of the staff was found to have good knowledge and favorable attitudes towards PBL |
| 2015 | Al-Drees | Saudi Arabia | Asia | 510 | Students | Survey or questionnaire | None | Not specified | Knowledge retention | | A PBL hybrid curriculum helped students improve their knowledge and learning skills. Students and staff training is required before the utilizing PBL |
| 2015 | Al-Shaikh | Saudi Arabia | Asia | 52 | Students | Survey or questionnaire | None | Not specified | Knowledge retention | Social and communication skills | Students perceived PBL positively, especially for clinical knowledge, critical thinking, communication skills and interpersonal relations. |
| 2015 | Khan | Saudi Arabia | Asia | 92 | Students and tutors | Survey or questionnaire | None | Not specified | Student satisfaction | Tutor satisfaction | 50% tutors thought that PBL was better than lecture-based learning. Students appreciated PBL content but could not identify other benefits of PBL |
| 2015 | Hande | India | Asia | 464 | Students | Survey or questionnaire | None | Not specified | Knowledge retention | Social skills | PBL improved the students’ acquisition of knowledge, generic and work-related skills and attitudes |
| 2015 | Nosair | United Arab Emirates | Asia | 250 | Students | Survey or questionnaire | None | Not specified | Student satisfaction | - | PBL environment is generally perceived positively by the medical students |
| 2015 | González | Spain | Europe | 204 | Students | Survey or questionnaire | Traditional or lecture | Not specified | Performance | Student satisfaction | PBL graduates were satisfied with their competencies and felt that they were equally if not better prepared for practice than peers from traditional systems |
| 2015 | Amoako-Sakyi | Ghana | Africa | - | Not specified | Descriptive experience | None | The global curriculum | Not specified | - | In spite of its cost implication, a PBL curriculum can be successfully implemented in resource-constrained settings |
| 2016 | Yanamadala | United States of America | America (north) | 202 | Students | Survey or questionnaire | None | Geriatric | Performance | - | Assessment of the curriculum demonstrated that medical students achieved in-depth learning across multiple geriatric competencies |
| 2016 | Demirören | Turkey | Europe | 561 | Students | Descriptive experience | None | Not specified | Other | - | Medical students used self-learning skills and believed in their ability to learn effectively in the PBL context |
| 2016 | Chang | United States of America | America (north) | - | Students and tutors | Opinion, editorial, comment | None | Not specified | Tutor satisfaction | - | The feelings of the authors regarding PBL has been overwhelmingly positive despite potential areas of improvement and continued fine-tuning |
| 2017 | Balendran | India | Asia | 26 | Students | Comparative non-randomized study | Traditional or lecture | Forensic Medicine | Performance | - | Better scores in the PBL group manifested in recall, cognitive domains, co-operation, team work and problem-solving skills |
| 2017 | Chang | Taiwan | Asia | 94 | Students | Survey or questionnaire | Traditional or lecture | Obstetrics and gynecology | Performance | - | PBL groups had significantly better scores |
| 2017 | Tshitenge | Botswana | Africa | 81 | Students | Survey or questionnaire | None | Family Medicine, Internal Medicine, Pediatrics and Surgery | Student satisfaction | - | Students rated PBL process as "good" and PBL facilitation as "very good" |
| 2017 | Alduraywish | Saudi Arabia | Asia | 170 | Students | Survey or questionnaire | None | Not specified | Student satisfaction | - | Students were satisfied with their experience with PBL and improved their generic skills. Students agreed that there was a heavy workload on them |
| 2017 | Eltony | Egypt | Asia | 71 | Students | Descriptive experience | None | Patient safety education program | Knowledge retention | Student satisfaction | The patient safety education program within a PBL curriculum was positively perceived by students |
| 2018 | Zhang | China | Asia | 1487 | Students | Systematic review and meta-analysis | Traditional or lecture | Radiology | Performance | - | PBL is more effective on radiology education than traditional methods (including learning interest, scope of knowledge, team spirit and oral expression) |
| 2018 | Hincapié | Mexico | America (south) | 100 | Students | Comparative non-randomized study | Traditional or lecture | Biochemistry | Performance | - | PBL significantly increases the academic performance and the critical thinking level |
| 2018 | Mughal | Pakistan | Asia | 210 | Students | Descriptive experience | None | Not specified | Performance | Social skills | PBLs facilitate development of problem-solving skills’ and social skills and have less emphasis on cognitive skill development |
| 2018 | Yadav | Nepal | Asia | 113 | Students | Survey or questionnaire | None | Not specified | Student satisfaction | - | Student’s attitude toward PBL was positive PBL , considered effective in improving knowledge, problem-solving, self-directed learning and teamwork |
| 2019 | Ma | China | Asia | 1003 | Students | Systematic review and meta-analysis | Traditional or lecture | Pediatric | Performance | - | PBL is more effective on pediatric medical education than the traditional teaching method in improving theoretical knowledge, skills and case analysis scores |
| 2019 | Berger | Germany | Europe | 112 | Students | Comparative and randomized study | Traditional or lecture | Cardiopulmonary resuscitation | Performance | - | PBL (combined with simulation training) leads to a short-term increase in initiating sufficient CPR by medical students as compared to classical education |
| 2019 | Alquliti | Saudi Arabia | Asia | 101 | Students | Comparative non-randomized study | Traditional or lecture | Not specified | Performance | - | PBL curriculum students showed a significantly higher overall performance |
| 2019 | Yoo | Korea, South | Asia | 118 | Students and tutors | Survey or questionnaire | None | Not specified | Student satisfaction | Tutor satisfaction | The overall satisfaction scores for the PBL program were neutral |
| 2019 | Asad | Saudi Arabia | Asia | 120 | Students | Comparative non-randomized study | Traditional or lecture | Not specified | Student satisfaction | - | Students preferred PBL over lectures in a system based hybrid curriculum |
| 2019 | Hu | China | Asia | 74 | Students | Comparative and randomized study | Traditional or lecture | Endocrinology | Knowledge retention | Student satisfaction | The combination of flipped classroom and PBL is be a better option over lecture-based classroom but can increase students’ workload |
| 2019 | Thompson | Georgia | Europe | 213 | Students | Comparative non-randomized study | Traditional or lecture | Anatomy (Cadaveric Dissection) | Performance | - | Cadaveric dissections coordinated with PBL cases increased student self-confidence but did not improve examination scores |
| 2019 | Aldayel | Saudi Arabia | Asia | 259 | Students | Survey or questionnaire | None | Not specified | Student satisfaction | - | The students’ perception toward PBL was more positive than negative, but further improvements are needed (tutors should be trained to guide the PBL) |
| 2020 | Mpalanyi | Uganda | Africa | 18 | Students | Survey or questionnaire | None | Radiology | Student satisfaction | - | Most students agreed that the PBL curriculum was efficient and had met their expectations and desired objectives |
| 2020 | Li | China | Asia | 122 | Students | Comparative and randomized study | Traditional or lecture | Clinical laboratory | Performance | Student satisfaction | PBL resulted in significantly better theory test scores, better student feedback scores, and clinical performance assessments, and a higher rate of satisfaction |
| 2020 | Zhao | China | Asia | 354 | Students | Comparative and randomized study | Traditional or lecture | Thyroid surgery | Performance | - | PBL is an effective method for improving medical students performance and enhance their clinical skills |
| 2020 | Liu | China | Asia | 1817 | Students | Systematic review and meta-analysis | Traditional or lecture | Variable | Knowledge retention | Student satisfaction | Hybrid (PBL and lecture) pedagogy (compared with lectures) increased clinical knowledge, clinical skills, comprehensive ability and teaching satisfaction |
| 2020 | Margolius | United States of America | America (north) | 68 | Students | Survey or questionnaire | None | Not specified | Performance | - | The study illustrates from the students’ perspectives, the benefits of the PBL as an instructional method |
| 2020 | Korkmaz | Turkey | Europe | 354 | Students | Survey or questionnaire | None | Not specified | Student satisfaction | - | The overall satisfaction rate was high |
